# Initial Experience with the Safe Implementation of Transanal Total Mesorectal Excision (TaTME) as a Standardized Procedure for Low Rectal Cancer

**DOI:** 10.3390/jcm10010072

**Published:** 2020-12-28

**Authors:** Luca Dittrich, Matthias Biebl, Rosa Schmuck, Safak Gül, Sascha Weiss, Oliver Haase, Michael Knoop, Ibrahim Alkatout, Johann Pratschke, Felix Aigner

**Affiliations:** 1 Department of Surgery, Campus Charité Mitte and Campus Virchow-Klinikum, Charité-Universitätsmedizin, Corporate Member of Freie Universität Berlin, Humboldt-Universität zu Berlin, 13353 Berlin, Germany; matthias.biebl@charite.de (M.B.); rosa.schmuck@charite.de (R.S.); safak.guel@charite.de (S.G.); sascha.weiss@klinikum-barnim.de (S.W.); oliver.haase@charite.de (O.H.); michael.knoop@charite.de (M.K.); johann.pratschke@charite.de (J.P.); 2 Department of Gynaecology, Christian-Albrechts-University Kiel, 24103 Kiel, Germany; ibrahim.alkatout@uksh.de; 3 Department of Surgery, St. John of God Hospital Graz, 8010 Graz, Austria

**Keywords:** rectal cancer, mesorectal, transanal, laparoscopic, local recurrence, survival, conversion rate

## Abstract

Introduction: The laparoscopic approach for TME is proven to be non-inferior in oncological outcome compared to open surgery. Anatomical limitations in the male and obese pelvis with resulting pathological shortcomings and high conversion rates were stimuli for alternative approaches. The transanal approach for TME (TaTME) was introduced to overcome these limitations. The aim of this study was to evaluate the outcomes of TaTME for mid and low rectal cancer at our center. Methods: TaTME is a hybrid procedure of simultaneously laparoscopic and transanal mesorectal excision. A retrospective analysis of all consecutive TaTME procedures performed at our center for mid and low rectal cancer between December 2014 and January 2020 was conducted. Results: A total of 157 patients underwent TaTME, with 72.6% receiving neoadjuvant chemoradiation. Mean tumor height was 6.1 ± 2.3 cm from the anal verge, 72.6% of patients had undergone neoadjuvant chemoradiotherapy, and 34.2% of patients presented with a threatened CRM upon pretherapeutic MRI. Abdominal conversion rate was 5.7% with no conversion for the transanal dissection. Early anastomotic leakage occurred in 7.0% of the patients. Mesorectum specimen was complete in 87.3%, R1 resection rate was 4.5% (involved distal resection margin) and in 7.6%, the CRM was positive. The three-year local recurrence rate of 58 patients with a follow-up ≥ 36 months was 3.4%. Overall survival was 92.0% after 12 months, and 82.2% after 36 months. Conclusion: TaTME can be performed safely with acceptable long-term oncological outcome. Low rectal cancer can be well addressed by TaTME, which is an appropriate alternative with low conversion, local recurrence, adequate mesorectal quality and CRM positivity rates.

## 1. Introduction

Colorectal cancer is the third-most frequent cancer worldwide, with an incidence of up to 10.2% in Western population [1]. Low anterior rectal resection following the principles of total mesorectal excision (TME) is still the gold standard of surgical treatment for mid and low rectal cancer [2]. The quality of the TME specimen, as well as involved circumferential resection margin (CRM), have shown to be predictive for local recurrence and cancer-free survival [3,4,5].

Laparoscopic surgery in rectal cancer has proven beneficial regarding postoperative pain, blood loss, and wound infections, as well as recovery time [6,7]. Mid- and long-term oncological outcome of laparoscopic surgery is similar to open resection, with a 3-year locoregional recurrence rate of 5% each [8]. Nevertheless, high positive CRM (17.2%) and conversion rates of up to 16.0% in experienced centers are reported for laparoscopic surgery [8,9].

Transanal total mesorectal excision (TaTME) was introduced for mid and lower rectal cancer and is proposed to allow a precise mesorectal dissection through better visualization in anatomically limited pelvis (male, narrow, obese). The “bottom-up” approach has been proposed to improve TME quality (88.5% complete TME) with low local recurrence rates in specialized centers in the mid-term follow-up [10,11,12,13,14].

Despite these promising results, Norwegian and Dutch authors recently reported early TaTME experiences with an unacceptably high local recurrence rate of almost 10% within two years and consequently critically appraised the implementation of TaTME in low-volume centers without stringent proctorship in the early implementation phase. Additionally, these results prompted a national moratorium for TaTME in Norway, blaming technical failures for the often multifocal devastating recurrences [15].

Without doubt, it has turned out that the transrectal approach harbors specific challenges, which need to be addressed properly. Interestingly, the common practice of performing an abdomino-perineal excision for low rectal cancers has somehow mitigated the prevailing lack of anatomical specification of the fascial layers around the pelvic floor, and the transanal dissection very close to tumors adjacent to the sphincter apparatus with the aim of performing a continence preserving resection unmasked these limitations. Further, the new technique may also have resulted in hampered oncological prudence for very low or advanced cancers, due to the aim of performing a sphincter preserving operation in all technically feasible cases.

Recently, however, several high-volume centers as well as international registry data contradicted the Norwegian experience, highlighting the importance of a structured training for safe implementation and indication of this challenging new technique [10,16].

The aim of the present study is to report our single-center experience of implementation of TaTME for mid and low rectal cancer, including all learning curve cases and evaluate the merit of this procedure in the management of rectal cancer.

## 2. Methods

### 2.1. Study Design

This study was a retrospective analysis of all consecutive patients undergoing TaTME in our center as recorded in the prospectively maintained international TaTME registry from December 2014 to January 2020. The LOREC^®^ TaTME registry is a database collecting clinical and histopathological data of patients undergoing surgery with transanal assistance for benign and malignant diseases described as TaTME. All consecutive patients treated at our institution had been included prospectively in this database. All patients provided written informed consent for being treated with the new technique of TaTME, as well as for the Charité IRB-approved (Reg.-No. 711/16) data collection within the international registry and retrospective data analysis.

Patients data were entered in pseudonymized form by the clinical team including the following information:

- Patient data: sex, date of birth;
- Pre-operative information: tumor staging (CT/MRI), previous treatments (e.g., neoadjuvant treatment);
- Surgery specific data;
- Post-operative course;
- Long-term follow-up data (Complications—Clavien–Dindo; readmissions);
- Histopathological and oncological outcomes.

### 2.2. Surgical Technique

Patients were placed in the lithotomy position and prepped the usual way for rectal cancer surgery. Care was taken to ensure comfortable positioning of the legs in bootstraps and extensive padding within a vacuum mattress to enable firm immobility within the steep Trendelenburg position during surgery. Whenever possible, a two-team approach with an abdominal and a transanal team was used. When two teams worked together, the pneumoperitoneum was established first to prevent retroperitoneal air cushioning due to the sub-peritoneal air inflation during TaTME. Both the abdominal and the transanal team worked with a pressure of 14mmHg.

### 2.3. Abdominal Procedure

The abdominal cavity was entered through an umbilical access and, usually, a multiport approach was used. As the specimen was always extracted through the abdominal wall, the retrieval site was either the umbilicus, or—if performed—the ileostomy site in the right lower quadrant. First, the inferior mesenteric vein (IMV) was identified and mobilized from medial to lateral, with care taken to preserve the anterior layer of the renal fat capsule and not to mobilize the pancreatic tail. Next, the inferior mesenteric artery (IMA) was identified and mobilized. The mesocolic plane was identified from medial to lateral preserving the fascia of Told covering the left ureter. The IMV was clipped close to the inferior margin of the pancreas, and the left colic flexure was completely mobilized. The IMA was double-clipped and transected close to its origin from the aorta (high-tie), and care was taken to mobilize the surrounding lymphatic tissue without damage to the hypogastric nerve fibers. The greater omentum was dissected from the transverse mesocolon and the lesser sac entered starting from the level of the falciform ligament. The left colic flexure was taken down. To ensure maximum mobility of the colon, the left-sided transverse mesocolon was completely mobilized, and transected close to the pancreas from lateral to medial close to the first left-sided branches of the middle colic vessels. The TME planes were completed in a circular fashion. Anteriorly, the peritoneum was only incised at the lowest point of the cul-de-sac, and the circular dissection pursued down to the level of S3.

### 2.4. Transanal Phase

#### 2.4.1. Prepping of the Transanal Access

After sterile prepping, a LoneStar (CooperSurgical, Trumbull, CT, USA) retractor was put in place and the anal sphincter carefully dilated. If the tumor was located more than 2–3cm above the dentate line, the transanal port (GelPoint Path, Applied Medical) was introduced, and two wet swaps placed inside the rectum below the tumor. The transanal insufflation using the AirSeal System (CONMED, Utica, NY, USA) was initiated. The first step was a safe purse string suture using a monofilament 2–0 suture below the swap to tightly close the rectum with the tumor. The insufflation was terminated, the port opened and the purse string suture closed tightly. Next, the distal rectal stump was rinsed with iodine-saline solution. Following re-insufflation of the rectal stump, the rectum was transected using the monopolar cautery hook, and the TaTME procedure started.

In cases of lower tumors, the anal canal was dilated and a conventional anal canal retractor introduced. After positioning of a swap inside the rectum to avoid any spilling, and making sure to maintain at least a 1cm distal margin to the tumor, the rectum was cut with monopolar energy and, simultaneously, the purse string suture was set, making sure to completely evert the tumor bearing inner part of the rectum and firmly close the purse string. A second purse-string suture was set if first was insufficient. In case of very low rectal cancers (iuxtasphinteric), frozen sections from the resection surface or the external part of the rectum were performed to ensure tumor-free distal and circumferential margins (R0). After that, the transanal port was put in place and the space rinsed with iodine/saline solution. During the whole procedure, any compromise to the tightness of the purse string was immediately corrected by a second purse string sutures.

#### 2.4.2. Transanal Resection

Following transection of the rectum, the levator muscle was identified at the dorsal circumference covered by the endopelvic fascia. Following the transsection layer to the anterior aspect, the rectum was completely mobilized and the caudad beginning of the mesorectal plane identified. Care was taken to leave the deep pelvic fascia covering the levator muscle, and to spare the fibrous tissue dorsal to the urethra on the ventral side. In a screwed circumferential way, the dissection was driven cranially to the abdominal and transanal rendezvous. Transanal preparation was performed using the monopolar hook and applying the traction-countertraction principle to expose the mesorectal dissection planes. Following the rendezvous (typically first on the ventral 12 o’clock position, then on the dorsal 6 o’clock position), the lateral transection was performed in collaboration between the abdominal and the transanal team.

The specimen was harvested through the abdominal wall, and depending transection level, either a transanal stapled or a hand-sewn transanal side-end coloanal anastomosis was performed. Hand-sewn anastomoses were performed with interrupted sutures (3–0 absorbable polyfilament thread) in side-to-end or end-to-end fashion. Stapled anastomoses were performed using a 29 or 33mm circular stapler in side-to-end or end-to-end fashion.

All patients were transanally drained for 48 h, and a transabdominal drain was placed in the pelvis for 48 h.

### 2.5. Patients

The potential indication for a TaTME approach was given if a TME for mid/low rectal cancer (at or below 12cm from the anal verge (AV)) was indicated by our multidisciplinary tumor board. While early in the experience, all TME-patients were evaluated for a transanal approach, we later switched to an anatomy driven approach, allocating patients low rectal cancer (<6cm from the AV), and patients with a bulky tumor or a narrow and/or deep pelvis to a TaTME procedure.

Both male and female patients were included. Patients were included independent of their T stage (mrT1-T4) and 65.8% of the study population were diagnosed preoperatively with positive lymph nodes (mrN+). Thoraco-abdominal computed tomography (CT) and MRI-scan of the lower abdomen and pelvis were routinely performed preoperatively to stage rectal cancer patients.

Tumor recurrence up to 180 days after diagnosis were defined as synchronous cancer occurrence, and, therefore, listed as preoperative M+ stage.

### 2.6. Statistical analysis

Data were reported as mean +/− standard deviation or total numbers (%). Intergroup comparisons were conducted using the Chi-2 test for dichotomous variables, and the student t-test for parametric numeric, or the Mann–Whitney U test for non-parametric numeric variables. Normal distribution was determined using the Kolmogorov–Smirnov test. Survival analyses were conducted using the Kaplan–Meier method and the log-rank test. For all analysis, a *p*-value of equal or below 0.05 was considered statistically significant.

## 3. Results

Between December 2014 and January 2020, 157 consecutive patients with mid or low rectal cancer underwent combined procedure (laparoscopic and transanal) for TME at our institution. A total of 317 patients underwent surgical treatment for rectal cancer between 2014 and 2020, in 157 (49.5%) patients TaTME was performed. A total of three patients (1.9%) had metastatic disease at time of initial diagnosis (synchronous liver metastasis). A total of seven (4.5%) patients were staged with T4 in the pre-treatment MRI. Involvement of the mesorectal fascia of less than 1mm was suspected in 54 (34.2%) patients. The preoperative patient characteristics are listed in Table 1.

The mean tumor height was 6.1 cm and 59.2% of the tumors were located at 6 cm or below from the AV. Neoadjuvant treatment had been performed in 72.6% of the patients.

### 3.1. Intraoperative Data

Intraoperative data are displayed in Table 2. In total, 85.4% of all surgeries were performed in a simultaneous two team-approach (i.e., at least the rendezvous procedures between abdominal and transanal part were done simultaneously). Laparoscopic abdominal dissection was performed in 98.1%. The abdominal conversion rate was 5.7%. All conversions were due to medical reasons (morbid obesity, CO_2_ retention, adhesions) for the abdominal part, no conversion was necessary for the transanal dissection. Mean anastomotic distance from the AV was 3.5cm. The majority of the anastomoses were stapled and, in seven patients, TaTME was primarily performed as low Hartmann procedure without anastomosis. In 86%, a defunctioning loop ileostomy was created and, in 10.2%, no stoma was created. A total of 3.8% of the patients were resected without reconstruction with creation of a terminal colostomy. No urinary tract (ureter/urethra) injury occurred.

### 3.2. Postoperative Outcome

Postoperative outcomes are listed in Table 3. In-hospital and 30-day overall complication rate was 31.2%. A total of twenty-one patients (13.4%) required a re-operation. In total, four patients were re-operated due to ischemia of the colon with preservation of the anastomosis in one patient and permanent deviation in three patients. A total of two patients were operated due to small bowel obstruction; one due to an internal herniation, the other due to stenosis at the ileostomy site, resulting in early stoma closure with healed colorectal anastomosis.

In 11 patients (7.0%), an early anastomotic leakage (defined as occurring within 30 days postoperatively) was observed. Four anastomoses successfully healed under endoluminal VAC therapy (36.4%) and seven patients (63.6%) required re-operation, five of them after initial endoluminal VAC therapy for damage control. A total of three patients (27.3% of patients with leakage and 1.9% of the overall study population) were discharged with a permanent colostomy. In total, ten of eleven (90.9%) patients with anastomotic leakage underwent TaTME with planned defunctioning stoma.

**Figure 1:**
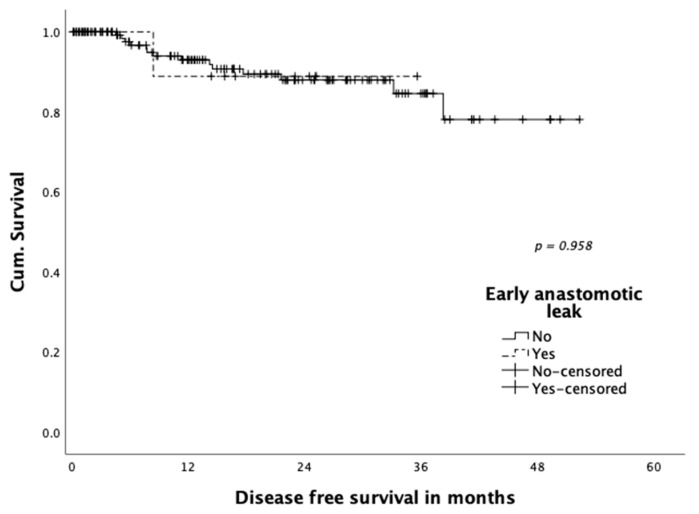
Disease-free survival according to early anastomotic leakage.

**Figure 2:**
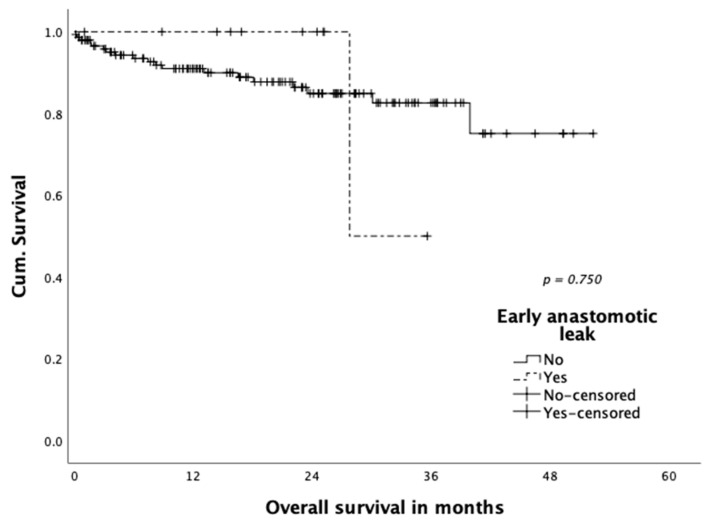
Overall survival according to early anastomotic leakage.

Neither disease-free survival (Figure 1) nor overall survival (Figure 2) were shown to be influenced statistically significantly by occurrence of anastomotic leakage (*p* = 0.958 and *p* = 0.750, respectively). A total of three patients (1.9%) died within 30 days: One patient died on postoperative day four in the course septic multiple organ failure after reoperation for colonic ischemia. One patient died on postoperative day eight due to a myocardial infarction. The third patient died 15 days post-operation with an ischemic brain damage after cardiogenic shock and resuscitation due to cardiac comorbidities.

**Figure 3:**
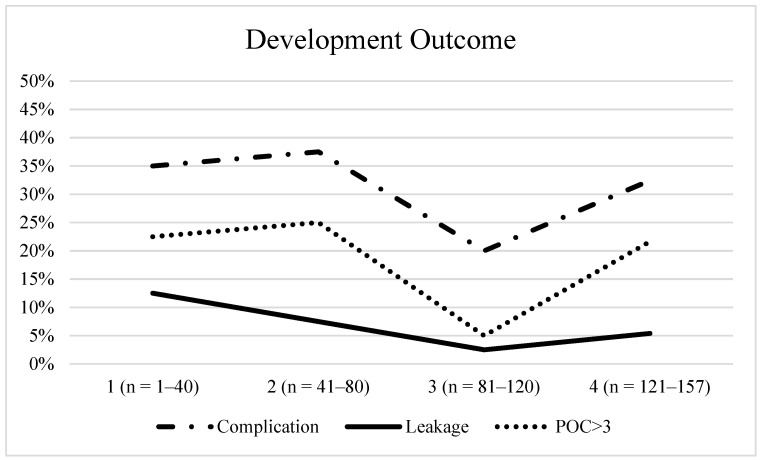
Development Outcome.

Regarding the learning curve, we divided our patient cohort into four equal groups according to case number (e.g., 1: n = 1–40, 2: n = 41–80, etc.) and analysed overall and severe complication rates as well as leakage rates (Figure 3). Over time, we recognized a decrease in complications and anastomotic leak rates, which, however, did not reach statistical significance. The data point to a flat and long learning curve due to the challenging technique.

### 3.3. Histopathological Outcome

Mean CRM was 14.5 mm and, in 7.6%, the CRM was positive (Table 4). Positive marginal status did not differ between patients with low rectal tumors (<6cm) (7.6%) and tumors >6cm from anal verge (7.7%). Complete pathological response (pCR) following chemoradiation was detected in 19.3% (ypT0), with a mean number of harvested lymph nodes of 16.2. In 87.3% of the specimens, mesorectal quality was reported as complete (Mercury grade 1) and R1 resection rate was 4.5% (involved distal resection margin).

### 3.4. Oncological Outcome

The oncological outcome is shown in Table 5.

**Figure 4:**
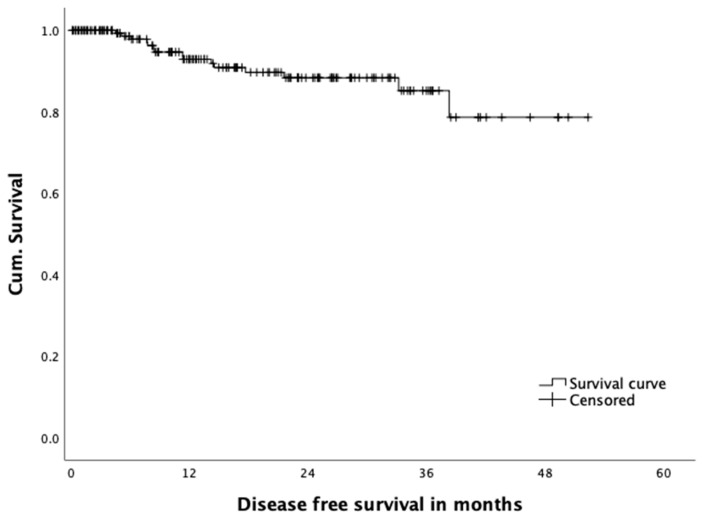
Overall disease free survival.

Figure 4 shows an overall disease-free survival after 12 months of 92.2%, and 85.2% after 36 months, respectively. After a mean follow-up of 19.5 months, we recorded a local recurrence rate of 3.2% in our patients, with an actual three-year local recurrence rate of 3.4% in 58 patients with a follow-up exceeding 36 months.

**Figure 5:**
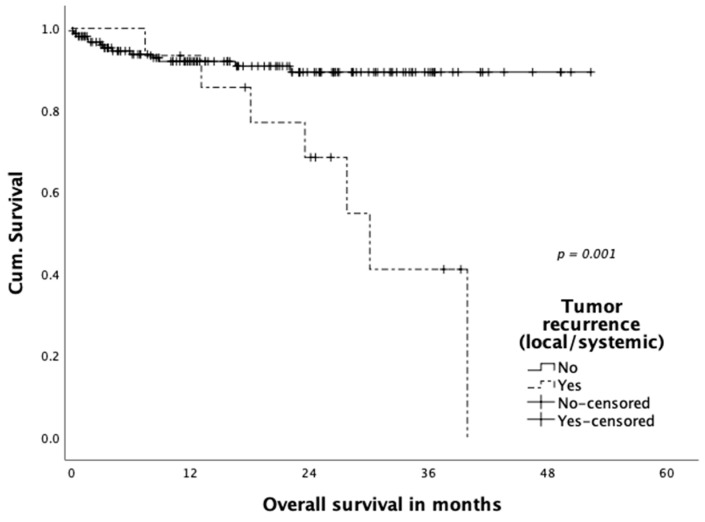
Overall survival according to tumor recurrence.

**Figure 6:**
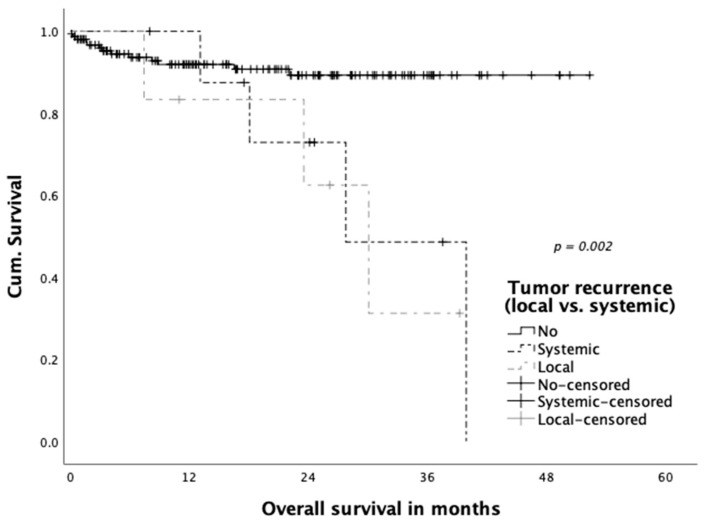
Overall survival according to site of tumor recurrence (local/systemic).

Three patients (1.9%) were diagnosed with simultaneous local and systemic recurrence and further three patients (1.9%) with only local recurrence in the follow-up. A total of thirteen (8.3%) patients developed distant metastatic disease. Mean time to the occurrence of local and systemic recurrence did not differ significantly (14.2 vs. 13.9 months). Tumor recurrence significantly impacted overall patient survival (local and systemic) and is displayed in Figure 5 (36-month survival rate 41.1% in patients with tumor recurrence versus 89.3% without recurrence (*p* = 0.001)). The impact of local recurrence versus systemic recurrence was compared separately in Figure 6, with a significantly reduced overall survival in patients with both local as well as systemic tumor recurrence (*p* = 0.002).

**Figure 7:**
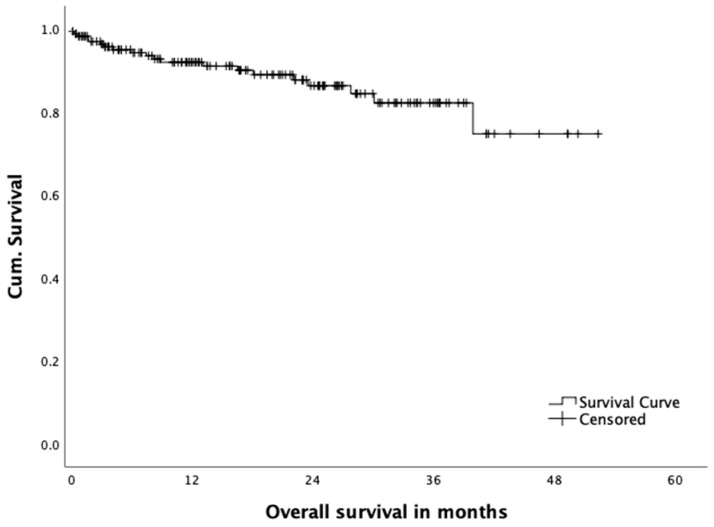
Overall survival.

A total of fourteen percent of the patients died within the observational period. A total of 6.3% of the deaths were cancer related, 2.5% were not cancer related, three patients (1.9%) died postoperatively (30-day mortality) and, in 3.2%, cause of death could not be determined. Overall survival is shown in Figure 7. Overall survival was 92.0% after 12 months and 82.2% after 36 months.

## 4. Discussion

The concept of a transanal approach to rectal cancer treatment was first developed from a NOTES perspective, using the anus as an access route [17]. This concept did not flourish in terms of reducing the abdominal access trauma; however, it improved the dissection of the lower third of the rectum close to the pelvic floor by direct visualization of the structures and the ability to access the supralevatoric rectum through a direct, straight access. The concept was evaluated with interest, as laparoscopic rectal surgery is equal to open surgery [7], but even in highly proficient centers, conversion rates, especially in low rectal cancer patients, are constantly reported to be around 15% [8]. Furthermore, the rates of “technical” excision of the sphincter in patients, who could have received a continence-preserving procedure are ill documented, but may range up to one out of four patients with low rectal cancer. Therefore, irrespective of other technical developments such as improvements in surgical instruments or robotic systems in transabdominal colorectal surgery, the access from below and the possibility of not having to exceed the tumor in the rectum in order to reach the projected anastomotic region is appealing. It is able to work under constant direct control of the margins directly on the pelvic floor and the sphincter complex. This defines the merits and potential benefits of such an access. This is not only relevant for the correct oncologic treatment of low rectal tumors, and the chance to prevent a definitive colostomy, but also to potentially better preserve nerval function and, thus, maintain quality of life in this patient cohort. According to our own data derived from the first 50 TaTME cases, LARS Score after 12 months was 27 (minor LARS; *n* = 39). 24 months after surgery a median LARS score of 14 (no LARS; *n* = 34) was assessed, highlighting a significant reduction (*p* < 0.001) due to pelvic floor exercise and transanal irrigation. However, only scarce prospective data about non-inferiority pelvic floor function and quality of life of TaTME patients in comparison to a conventional abdominal approach are available so far [18,19,20].

Furthermore, in 6.4%, a purse string failure was recognized intraoperatively and a second purse string was set immediately. This shortcoming and technical failure was reported in the TaTME community and recommendations for a second purse string suture was highlighted in the COLOR III study protocol amendment [21]. Regarding the oncological outcome, we did not detect any local recurrences in patients with purse string failure in our study group.

Surgical and oncological outcome of TaTME for low and mid rectal cancer was demonstrated to be equal compared to conventional TME (laparoscopic and open) in a meta-analysis of observational and matched-paired cohort studies [14]. However, data from randomized controlled trials (COLOR III and ETAP-GRECCAR 11 trials) are still missing to correctly answer this question of non-inferiority [21,22]. Significant difference was reported in the conversion rate to the open approach between TaTME and laparoscopic TME (1.4% vs. 8.8%; OR 0.17; 95% CI 0.1–0.29, *p* < 0.00001) [14], which was nil in our cohort.

Simillis et al. could not demonstrate any significant difference in local recurrence rate, disease-free survival and overall survival in the 5-year-follow-up in a network meta-analysis including all TME techniques (open, *n* = 2350; lapTME, *n* = 3276; robotic, *n* = 561; TaTME, *n* = 50) [23].

Significant superiority of TaTME, however, was shown in the rate of negative CRM, which was confirmed by Hajibandeh et al. (OR 0.67; 95% CI 0.45–0.98, *p* = 0.04) [14].

The published data reflect the TaTME pioneering centers‘ experience, also including cases from the beginning of the learning curve. A selection bias, however, with inclusion of cases with accumulation of risk factors for inappropriate TME cannot be disclaimed. Those were exclusively excluded in comparing studies like COLOR II [8,10,13,16,24,25,26].

Respecting this, local recurrence rates like those in our own series (3.2% at 3-year follow-up) after overcoming the learning curve are remarkable and comparable with 2–3-year follow-up data of the laparoscopic arms in the ACOSOG Z6051 trial (4.5%) and the COLOR II trial (5%) [8,27].

Worrying data from Norway and the Netherlands regarding multifocal local recurrences in the short-term follow-up (7.4 and 10%) significantly higher than those from national and international controls (2.4 and 1.8%) resulted in critical appraisals facing results of experienced high-volume centers with regard to center and selection bias and questioning inclusion of learning curve cases [25]. We think that our series of consecutive patients undergoing TME for mid and low rectal cancer clearly demonstrates the flat learning curve of the transanal approach even in experienced hands and a distinguished minimally-invasive institution. Additionally, we have entered all patient data into the international TaTME registry [28] for transparency and ongoing quality control.

The transanal approach inevitably results in a very low anastomosis within less than 2cm of the dentate line and, therefore, in our opinion, is not suitable as a “one-fits all” approach to mid and low rectal cancer surgery. However, in this series, it has proven to be a very versatile approach to the pelvic floor and allowed for safe dissection of this area in all patients, as documented by our 0% conversion rate for the transanal part of the procedure. Therefore, irrespective of the minimally invasive technique used for the abdominal phase of the operation, we think the technique has earned its merits for every situation, when the superior approach is limited or compromised by anatomical or tumor-related specific findings. With regard to the published problems of tumor-spread through the direct preparation, it needs to be reiterated that (1) a complete seal of the tumor site from the subperitoneal dissection plane, where the insufflation device (AirSeal^®^) results in a constant circulatory flow, and (2) no alteration of the oncologic understanding of proper resection margins, as they were implemented in rectal surgery decades ago, are absolutely key to performing a safe TaTME and strictly need to be adhered to. Unfortunately, as in many innovative techniques, the transanal approach for rectal cancer surgery is also threatened to be compromised by a too-quick distribution and too-loose indication by the surgical community.

Consequently, colorectal societies in many countries have raised concerns about structured training curricula due to the complexity of TaTME and fostered proctorship for the first own TaTME cases [25,29,30] to encounter technical pitfalls of the transanal approach (e.g., urethral injury or unfavourable oncological results due to technical failures like insufficient purse-string sutures). In those centers continuing the TaTME programme beyond 45 cases, local recurrence rates leveled off below 4% comparable to the rate we have observed in our series of >150 cases. In our center, the first 100 cases of TaTME were performed by the same team of leading surgeons (F.A. and M.B.) to sustain quality after having overcome the flat learning curve.

International recommendations aim at a minimum of 25 annual cancer resections by TaTME following the indications for benign or malignant rectal resection where there is anticipated technical difficulty in pelvic dissection; ideally reaching >40 annual resections involving the rectum for benign and/or malignant disease [31]. The high-volume of rectal cancer cases is therefore decisive since potential training TaTME cases are vanishing if indications for TaTME are restricted (e.g., not including high rectal cancer, T4 or sphincter involving or early rectal cancer).

TaTME is, therefore, reserved for sub-specialized colorectal institutions and should be selectively applied for patients with mid and low rectal cancer. After completion of a structured training programme, TaTME is a helpful alternative in cases where supraanal or intersphincteric resection are planned and, thus, performed by colorectal specialists, including cases where conversion and salvage strategies (APE) are discussed. 

## 5. Conclusions

Hybrid laparoscopic and TaTME is technically challenging with additional staff requirement especially when performed synchronously. TaTME shows a flat learning curve; however, it is proposed as a relevant alternative not only in mid rectal cancer but especially in low rectal cancer cases with anatomical limitations. In our study cohort, we observed low conversion rates— respectively, none in the transanal part as well as a low local recurrence rates.

## Figures and Tables

**Table 1:** Patient characteristics (*n* = 157).

Age (y); mean ± SD	60.6 ± 12.4
Sex; *n* (%)	
Male	117 (74.5)
Female	40 (25.5)
BMI (kg/m^2^); mean ± SD	26.2 ± 5.0
ASA classification; *n* (%)	
ASA 1	14 (8.9)
ASA 2	104 (66.2)
ASA 3	35 (22.3)
ASA 4	4 (2.5)
Tumor height AV (cm); mean ± SD	6.1 ± 2.3
Tumor height AV; *n* (%)	
>6cm	64 (40.8)
≤6cm	93 (59.2)
Neoadjuvant treatment; *n* (%)	
Yes	114 (72.6)
No	43 (27.4)
Preoperative T stage; *n* (%)	
mrT0	1 (0.6)
mrT1	5 (3.2)
mrT2	36 (22.9)
mrT3	100 (63.6)
mrT4	7 (4.5)
mrTx	8 (5.1)
Preoperative N stage; *n* (%)	
mrN-	42 (26.8)
mrN+	104 (66.3)
mrNx	11 (7.0)
Preoperative M stage; *n* (%)	
M0	137 (87.3)
M1	20 (12.7)
Preoperative mrCRM+ *; *n* (%)	54 (34.2)

ASA, American Society of Anesthesiologists; AV, Anal verge; BMI, Body mass index; CRM, Circumferential resection margin; mrTNM, TNM stage on preoperative MRI; SD, Standard deviation; * Positive CRM on MRI is defined as the distance of tumor or malignant lymph node to the mesorectal fascia of ≤1 mm.

**Table 2:** Intraoperative data (*n* = 157).

Operative time (min); mean ± SD	306.6 ± 108.5
Two-team approach; *n* (%)	134 (85.4)
Abdominal dissection; *n* (%)	
Open	3 (1.9)
Laparoscopic	154 (98.1)
Conversion; *n* (%)	
Abdominal	9 (5.7)
Perineal	0 (0.0)
Defunctioning stoma; *n* (%)	
None	16 (10.2)
Ileostomy	135 (86.0)
Colostomy	6 (3.8)
Anastomotic technique; *n* (%)	
None	7 (4.5)
Hand-sewn	54 (34.4)
Stapled (circular)	96 (61.1)
Anastomotic distance from AV (cm); mean ± SD	3.5 ± 1.5
Urinary tract trauma; *n* (%)	0 (0.0)
Pursestring failure; *n* (%)	10 (6.4)

AV, Anal verge; SD, Standard deviation.

**Table 3:** Postoperative Outcome (*n* = 157).

Complications; *n* (%)	49 (31.2)
Anastomotic leak	11 (7.0)
Colon ischemia	5 (3.2)
Compartment syndrome	1 (0.6)
Haemorrhage	2 (1.3)
Internal hernia	1 (0.6)
Obstruction	2 (1.3)
Perforation	1 (0.6)
Stoma complication	5 (3.2)
Wound breakdown	14 (8.9)
Cardiovascular complication	3 (1.9)
DVT	1 (0.6)
PE	2 (1.3)
Pulmonary complication	4 (2.5)
Renal Failure	7 (4.5)
Urinary tract infection	3 (1.9)
Others	3 (1.9)
Re-operation	21 (13.4)
Early anastomotic leak *; *n* (%)	11 (7.0)
Endoscopic therapy	9 (81.8)
Re-operation	7 (63.6)
Definitive stoma after leakage	3 (27.3)
Length of stay (days); mean ±SD	11.4 ± 9.2
Surgical morbidity (Clavien–Dindo III-V) **; *n* (%)	30 (19.1)
Postoperative death	3 (1.9)

AV, Anal verge; SD, Standard deviation; * Early anastomotic leak is defined as the overall anastomotic failure within the first 30 postoperative days. ** Surgical morbidity (Clavien–Dindo III-V) is defined as the overall morbidity/mortality within the first 30 postoperative days.

**Table 4:** Histopathological outcome (*n* = 157).

Tumor size (mm); mean ± SD	27.5 ± 17.4
Distal margin (mm); mean ± SD	21.0 ± 22.0
Circumferential margin (mm); mean ± SD	14.5 ± 11.4
Positive circumferential margin *; *n* (%)	12 (7.6)
Positive circumferential margin; *n* (%)	
Tumor height from AV >6cm, (*n* = 65)	5 (7.7)
Tumor height from AV ≤6cm, (*n* = 92)	7 (7.6)
No. lymph nodes harvested; mean ± SD	16.2 ± 6.3
pTMNT; *n* (%)	
T0	26 (16.6)
T1	17 (10.8)
T2	55 (35.0)
T3	52 (33.1)
T4	6 (3.8)
Tx	1 (0.6)
pTMNN; *n* (%)	
N0	112 (71.3)
N1	30 (19.1)
N2	15 (9.6)
Quality of mesorectal specimen (Mercury grade); *n* (%)	
I (complete)	137 (87.3)
II (nearly complete)	12 (7.6)
III (incomplete)	3 (1.9)
Missing	5 (3.2)
Resection margin R1; *n* (%)	7 (4.5)

AV, Anal verge; SD, Standard deviation; * Positive circumferential margin is defined as the distance of tumor or malignant lymph node to the mesorectal fascia of ≤1 mm.

**Table 5:** Oncological outcome (*n* = 157).

Follow-up (mo); mean ± SD (range)	19.5 ± 13.5 (0.1–52.3)
Local recurrence, *n* (%)	6 (3.8)
Local recurrence only	3 (1.9)
Simultaneous local/systemic recurrence	3 (1.9)
Tumor recurrence (systemic), *n* (%)	13 (8.3)
Death, *n* (%)	22 (14.0)
Cancer	10 (45.5)
Not cancer related	4 (18.2)
30-day mortality	3 (13.6)
Unknown	5 (22.7)
3-year follow up * (*n* = 58)	
Local recurrence, *n* (%)	2 (3.4)
Local recurrence only	0 (0.0)
Simultaneous local/systemic recurrence	2 (3.4)
Tumor recurrence (systemic), *n* (%)	7 (12.1)
Death, *n* (%)	12 (20.3)
Cancer	7 (58.3)
Not cancer related	2 (16.7)
30-day mortalityUnknown	1 (8.3)2 (16.7)

SD, Standard deviation; * Patients with a complete 3-year follow-up.

## Data Availability

Due to the principals of this research, participants of this study did not agree that their data is shared publicly, so supporting data is not available. For further information please contact the corresponding authors (L.D.; F.A.).
